# Manual of Chemistry

**Published:** 1899-07

**Authors:** 


Manual of Chemistry. A Guide to Lectures and Laboratory Work for Beginners in Chemistry. A Text-book specially adapted for Students of Pharmacy and Medicine. By W. Simon, Ph. D., M. D., Professor of Chemistry and Toxicology, College of Physicians and Surgeons, Baltimore; Professor of Chemistry in the Maryland College of Pharmacy. New (Sixth) edition. In one 8vo. volume of 532 pages, with 46 engravings and 8 colored plates illustrating 64 of the most important chemical tests. Price, cloth, $3 net. Lea Brothers & Co., Publishers, Philadelphia and New York.

This work has already proven its popularity, and the present issue shows considerable improvement over the other editions. As heretofore, the subject has been divided into seven parts, which contain so much of the matter under consideration as is believed to be necessary for a fair understanding of the subject, at the same time care has been taken to place in foreground all facts and data which are of direct interest to the physician. The seventh or last part, giving some of the principal facts of physiological chemistry, is of especial value to students of medicine particularly; the chapters on urinary analysis are of more than ordinary interest, they far surpass the ordinary works on this subject.

The author is not new in the field of chemistry, he is well known for his former efforts in this line. We can highly commend this work, not only for its valuable contents, but for its arrangement. Every doctor could profit by having this work in his library, and we know he would refer to it more frequently than to others of this line. W. R. N.

